# Association of tumour and stroma PD-1, PD-L1, CD3, CD4 and CD8 expression with DCB and OS to nivolumab treatment in NSCLC patients pre-treated with chemotherapy

**DOI:** 10.1038/s41416-020-0888-5

**Published:** 2020-05-20

**Authors:** Anna-Larissa Nadia Niemeijer, Sara Sahba, Egbert Frederik Smit, Birgit Ilja Lissenberg-Witte, Adrianus Johannes de Langen, Erik Thunnissen

**Affiliations:** 1 Department of Pulmonology, Amsterdam UMC, Vrije Universiteit Amsterdam, De Boelelaan 1117, 1081 HV Amsterdam, Netherlands; 2 Department of Thoracic Oncology, Netherlands Cancer Institute, Plesmanlaan 2, 1066 CX Amsterdam, Netherlands; 3 Department of Epidemiology and Biostatistics, Amsterdam UMC, Vrije Universiteit Amsterdam, De Boelelaan 1117, 1081 HV Amsterdam, Netherlands; 4 Department of Pathology, Amsterdam UMC, Vrije Universiteit Amsterdam, De Boelelaan 1117, 1081 HV Amsterdam, Netherlands

**Keywords:** Non-small-cell lung cancer, Prognostic markers

## Abstract

**Background:**

Immune checkpoint inhibitors are most beneficial in patients with high tumour PD-L1 expression. However, the use of PD-L1 expression is not straightforward. We investigated PD-L1 expression and immune cell (IC) infiltrates in non-small-cell lung cancer (NSCLC) patients treated with nivolumab.

**Methods:**

Tumour tissue specimens of 139 NSCLC patients were scored for tumour/stromal PD-L1 and various IC expression markers, and associated with durable clinical benefit (DCB) and overall survival (OS).

**Results:**

Median OS was higher for patients with high stromal infiltration of CD8^+^ ICs (9.0 months) compared with patients with low and intermediate infiltration (both 5.0 months, *p* = 0.035) and for patients with high infiltration of stromal CD4^+^ ICs (9.0 months) compared with patients with low and intermediate infiltration (both 5.0 months, *p* = 0.010) and this was confirmed in the validation cohort. Post hoc analyses showed that biopsies taken after the last line of chemotherapy (ACT) were predictive for DCB and OS, whereas samples obtained before the last line of chemotherapy (BCT) were not.

**Conclusions:**

Stromal infiltration of ICs can predict response to PD-1-directed immunotherapy in NSCLC patients. Interestingly, we found differences in the predictive value of IC markers between the ACT and BCT biopsies, suggesting that chemotherapy might influence the immune microenvironment.

## Background

The treatment of non-small-cell lung cancer (NSCLC) has been revolutionised by the introduction of antibodies targeting immune checkpoints, including those directed against the programmed cell death 1 (PD-1) or its ligand (PD-L1).^1^ PD-1 is expressed on activated B- and T cells and inhibits their functioning upon binding to PD-L1, which is expressed by tumour cells and a subset of immune cells.^2,3^ Nivolumab, an anti-PD-1 checkpoint inhibitor, has clinical activity in patients with NSCLC, and is effective as second-line therapy in patients with advanced-stage NSCLC.^4–6^ Unfortunately, only ~20% of patients experience a durable response to single-agent nivolumab treatment.^4,5^

Despite promising efforts on tumour mutational burden, PD-L1 expression is still the only clinically available biomarker.^7,8^ Nonetheless, the value of tumour PD-L1 expression as a predictive biomarker is not straightforward. There are multiple assays with varying definitions of biomarker positivity, and in patients with the highest expression PD-L1 levels, a positive immunohistochemistry (IHC) test defined by ≥50% tumour PD-L1 expression, the response rate is only ~45%, while those with completely negative (<1%) tumour PD-L1 staining still have a response rate of ~10%.^9–11^

There is increasing evidence that immune contexture, i.e., infiltration of tumour tissue by immune cells, plays an important role in the sensitivity of cancers to respond to immune checkpoint inhibitor therapy.^12,13^ Most studies that examined the immune contexture used a mixed cohort of solid cancer patients receiving immune checkpoint inhibitors. Although immune cell PD-L1 expression and CD8^+^ infiltration are mentioned as markers correlated with (durable) response, none of the studies agree which predictive marker can be used in NSCLC patients.^13–15^ Therefore, the exact role of these specific immune cells in tumour inflammation and antitumour response to checkpoint inhibition therapy in NSCLC remains uncertain.

We hypothesised that immunohistochemical analysis of the immune contexture beyond tumour PD-L1 expression has predictive value for response to PD-1 checkpoint inhibitor therapy. Using tumour biopsies obtained prior to nivolumab treatment, tumour PD-L1 expression and CD8^+^ tumour-infiltrating immune cell expression were assessed, as well as stromal PD-1^+^, PD-L1^+^, CD3^+^, CD4^+^ and CD8^+^ immune cell expression. Next, these parameters were associated with durable clinical benefit (DCB) and overall survival (OS) in patients with advanced-stage NSCLC that were treated with nivolumab.

## Methods

### Subjects and samples

Since the introduction of immunotherapy for NSCLC in The Netherlands, patient characteristics, as well as treatment outcome and adverse events, are being prospectively collected in a national lung cancer immunotherapy database for all patients with a pathologically confirmed diagnosis of advanced-stage NSCLC, who are treated with immune checkpoint inhibitors. From this database, we retrospectively selected patients treated with nivolumab (3 mg/kg Q2W) from August 2015 to January 2017 at the Amsterdam University Medical Centers, location: VU Medical Center (VUmc). For each eligible patient, we retrospectively obtained a suitable histological biopsy sample that contained sufficient tumour tissue (i.e., at least 100 tumour cells), and was preferably not decalcified before paraffin embedding. Tissue blocks were obtained prior to the start of nivolumab treatment, either before or after the last line of treatment with platinum- doublet chemotherapy. All tissue analyses were performed at the VUmc Department of Pathology. The protocol was approved by the Medical Ethics Committee in compliance with the local institutional review board regulations [protocol number: U2017.003]. Clinical parameters required for this study were retrospectively extracted from (electronic) patient records.

### IHC analysis

Detailed information about the immunohistochemistry staining is provided in the Supplementary Material (Supplementary Material and Supplementary Table 1).

### Scoring of tumour PD-L1 expression

Tumour PD-L1 scoring was performed according to the instruction manual of the qualitative immunohistochemical assay developed by Dako as a companion diagnostic tool for nivolumab [PharmDx] using the 28.8 antibody. Tumour PD-L1 expression levels were determined by observing complete circumferential or partial linear expression (at any intensity) of PD-L1 on the plasma cell membrane of viable tumour cells. In parallel, the pattern of staining in CD4-stained slides was evaluated and compared with PD-L1-stained slides in order to avoid false-positive assessment due to PD-L1-expressing macrophages. Assessment of expression levels was performed in sections that included at least 100 evaluable tumour cells. Heterogeneous PD-L1 staining was evaluated according to the instruction of the kit, by dividing the tumour areas into sections with equal amounts of tumour cells at a low magnification, and scoring the percentage of PD-L1-positive cells for each area separately. Next the percent positivity from each area was added and divided by the total number of areas.^16,17^ The percentage of stained tumour cells in the entire specimen was categorised into the following categories: <1%, 1–5%, 5–10%, 10–25%, 25–50% and ≥50%. Assessment of tumour PD-L1 expression, as well as of the other IHC parameters, was performed by a senior experienced thoracic pathologist (ET) and blinded for treatment outcome.

### Scoring of immune cell infiltrates

Immune cell infiltration was assessed by evaluating the presence of PD-L1^+^-, PD-1^+^-, CD3^+^-, CD4^+^- and CD8^+^-stained immune cells in stromal tissue and CD8^+^ immune cells in tumour tissue. For assessment of stroma only, the peritumoural stromal area was scored, because of its prognostic importance.^14,18^ The peritumoural stroma was defined as the stroma directly adjacent to tumour cell areas. The density of immune cell infiltrates in the stromal and tumour compartments was scored as no infiltration, low infiltration, intermediate infiltration and high infiltration according to Paulsen et al. and Donne et al.^19,20^ To ensure a more detailed categorisation, we added an extra category ‘very low infiltration' to the existing scale with occasionally immune cells present. In the case of heterogeneous infiltration, the highest identifiable infiltration category was scored.

### Data collection

Clinical data on patients’ gender, age at the start of treatment, performance score and tumour histology were extracted from the available databases. Baseline CT scan of thorax + /− abdomen had to be obtained within 6 weeks before the start of treatment. Response evaluation was performed using CT imaging every 6 weeks or, after a partial response was obtained, every 3 months according to the institutional treatment protocol.

### Treatment outcome

The primary outcome measure of this study was DCB defined as complete response (CR), partial response (PR) or stable disease (SD) by RECIST v1.1 for a duration of ≥6 months after the start of nivolumab treatment.^21^ All available CT scans were scored blinded for the IHC results. Patients who were diseased or deteriorated before the first response assessment, were designated as patients without DCB. According to these data, two treatment outcome groups, patients with DCB and without DCB, were defined.

### Statistics

Mean with standard deviation (SD) were used as descriptive statistics for data that were normally distributed. Median with interquartile range (IQR) were used for not normally distributed data. For differences between two unpaired groups, the chi-square test was used for dichotomous or ordinal variables, and the independent samples’ *t* test or Wilcoxon rank-sum test (when the data were not normally distributed) for continuous variables. In order to attain sufficiently large groups for analysis, selected categories of infiltration by immune cells were combined based on group size (see Supplementary Table 2).

The following categories were combined, based on previous studies: stromal CD8^+^ and tumour CD8^+^ immune cell infiltration, stromal PD-1^+^ and CD8^+^ immune cell infiltration and stromal PD-L1^+^ and CD8^+^ immune cell infiltration.^14,15,22,23^

The final dataset was 1:1 randomised into a derivation cohort and a validation cohort at random by SPSS. The derivation cohort was used to examine the association between individual or combined IHC variables and DCB using univariate logistic regression models. The validation cohort was used to validate the observed associations between the study variables and DCB to nivolumab in a multivariate logistic regression model, built with a forward selection procedure (p entry < 0.1). OS was analysed using the Kaplan–Meier method and compared between two unpaired groups using log-rank tests. Hazard ratios (HRs) were estimated with univariate Cox proportional-hazard models for the expression levels of tumour and stromal markers with corresponding 95% confidence interval (CI). Statistical analyses were performed using SPSS, version 22 (IBM Corp., Armonk, NY). Figures were generated with GraphPad Prism, version 7.02. *P* values < 0.05 were considered to indicate statistical significance.

## Results

**Fig. 1:**
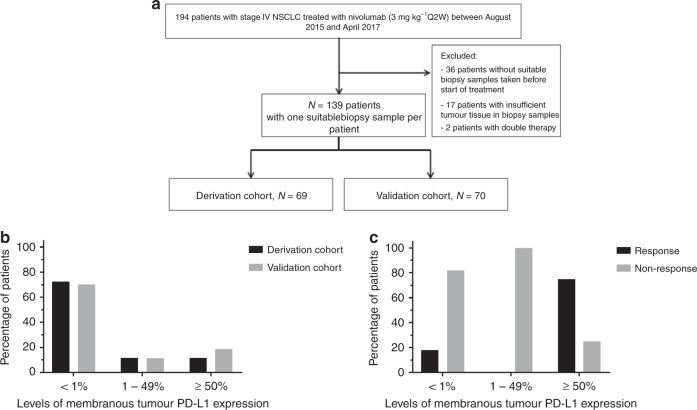
Flowchart of the study and distribution of PD-L1 and relation with durable clinical benefit (DCB). **a** Flowchart demonstrating the selection procedure of patient biopsy samples. **b** Distribution of tumour PD-L1 expression in both cohorts. In both cohorts, the largest proportion of patients had <1% tumour PD-L1 expression. **c** Tumour PD-L1 expression was associated with DCB in the derivation cohort.

In total, 194 patients were treated with nivolumab in the period of August 2015 till January 2017. Fifty-five patients were excluded because (1) no suitable biopsy samples were obtained prior to treatment (*n* = 36); (2) available biopsy samples did not contain sufficient tumour tissue (*n* = 17); (3) because patients were on combined therapy (tyrosine kinase inhibitors and immunotherapy) at the start of the treatment (*n* = 2) (Fig. 1a). Hundred-thirty-nine biopsy samples from 139 patients with advanced-stage NSCLC were included in this study. Patient and tumour characteristics for the derivation and validation cohorts are shown in Table 1. None of the baseline characteristics differed significantly among the cohorts. All patients received nivolumab as second-line treatment or beyond, after prior treatment with platinum-doublet chemotherapy + /− local treatment with radiotherapy or surgery. In the derivation cohort, 21.7% of patients showed DCB to nivolumab, versus 30.0% of patients in the validation cohort.

**Table 1:** Baseline characteristics.

	Derivation, *n* (%)	Validation, *n* (%)	*p* value
Number of patients	69	70	
Mean age in years, mean ± SD	62.3 ± 10.7	63.7 ± 7.2	0.37
Number of biopsy samples	69	70	0.87
Obtained after the last line of treatment (ACT)	36 (52.2)	35 (50.0)	
Obtained before the last line of treatment (BCT)	33 (47.8)	35 (50.0)	
*Sex*			0.61
Male	40 (58.0)	37 (52.9)	
Female	29 (42.0)	33 (47.1)	
*Histological subtypes*			0.31
Squamous	20 (29.0)	21 (68.6)	
Nonsquamous	46 (66.7)	48 (30.0)	
Other (adenosquamous, LCNEC, NOS or unknown)	3 (4.3)	1 (1.4)	
*ECOG performance score*			0.59
0	7 (10.1)	8 (11.4)	
1	44 (63.8)	40 (57.1)	
≥2	16 (23.2)	22 (31.4)	
Unknown	2 (2.9)	0 (0)	
*DCB rate*			0.34
Patients without DCB	15 (21.7)	21 (30.0)	
Patients with DCB	54 (78.3)	50 (70.0)	

### Tumour PD-L1 expression and stromal and tumour immune cell infiltration are associated with DCB and OS in the derivation cohort

The distribution of the levels of membranous PD-L1 expression in the derivation and the validation cohorts is visualised in Fig. 1b and described in Supplementary Table 3. The fraction of patients with <1% membranous PD-L1 expression in tumour tissue in the derivation and validation cohorts was 72.5% and 70.0%, respectively. Likewise, the fraction of patients with ≥50% membranous PD-L1 expression in the validation and derivation cohorts was 11.6% and 18.6%, respectively. For the category with membranous PD-L1 expression in tumour tissue between 1 and 49%, the fraction of patients in the validation and derivation cohorts was 14.3% and 11.4%, respectively (*p* = 0.62).

The results of univariate and multivariate analyses performed on the derivation cohort are shown in Table 2. In univariate analysis, tumour PD-L1 was associated with DCB, especially in the patients with ≥50% tumour PD-L1 expression (DCB rate 75%, OR 13.6, 95% CI 2.3–79.0) (Fig. 1c and Table 2). Multivariate analysis confirmed this association. This finding was subsequently evaluated in the validation cohort. The DCB rate was 39% in the validation cohort for tumour PD-L1 ≥ 50%, and tumour PD-L1 expression was not associated with DCB in the validation cohort (*p* = 0.63), see Supplementary Table 4.

**Table 2:** Results of univariate analysis of PD-L1, PD-1, CD8, CD4 and CD3 in relation to durable clinical benefit are shown for the derivation cohort.

Variables	Derivation cohort				
	DCB rates (%)	Univariate OR (95% CI)	*p* value	Multivariate OR (95% CI)	*p* value
*Tumour PD-L1 expression*			**0.014**		**0.041**
<1% (*n* = 50)	18	1.0		1.00	
1–49% (*n* = 8)	0	0		0.00	
≥50% (*n* = 8)	75	13.6 (2.3–79.0)		11.8 (1.7–79.7)	
*Stromal infiltration of PD-L1*^*+*^ *IC*			0.36		
Low infiltration (*n* = 40)	18	1.0			
Intermediate infiltration (*n* = 15)	33	2.4 (0.61–9.1)			
High infiltration (*n* = 9)	33	2.4 (0.47–11.8)			
*Stromal infiltration of PD-1*^*+*^ *IC*			**0.047**		
Low infiltration (*n* = 38)	13	1.0			
Intermediate infiltration (*n* = 21)	29	2.6 (0.70–10.0)			
High infiltration (*n* = 7)	57	8.8 (1.5–51.6)			
*Tumour infiltration of CD8*^*+*^ *IC*			0.11		
Low infiltration (*n* = 35)	14	1.0			
Intermediate infiltration (*n* = 18)	22	1.7 (0.40–7.4)			
High infiltration (*n* = 14)	43	4.5 (1.1–18.6)			
*Stromal infiltration of CD8*^*+*^ *IC*			0.063		
Low infiltration (*n* = 15)	7	1.0			
Intermediate infiltration (*n* = 21)	14	2.3 (0.22–24.9)			
High infiltration (*n* = 30)	37	8.1 (0.93–70.3)			
*Stromal infiltration of CD3*^*+*^ *IC*			**0.046**		
Low infiltration (*n* = 22)	5	1.0			
Intermediate infiltration (*n* = 21)	24	6.6 (0.7–61.9)			
High infiltration (*n* = 22)	41	14.5 (1.7–128.4)			
*Stromal infiltration of CD4*^*+*^ *IC*			**0.044**		
Low infiltration (*n* = 16)	6	1.0			
Intermediate infiltration (*n* = 13)	8	1.3 (0.071–22.1)			
High infiltration (*n* = 36)	36	8.5 (1.0–71.7)			
*Combined tumour and stromal infiltration of CD8*^*+*^ *IC*			**0.030**		0.073
Low infiltration of tumour and stromal CD8^+^ IC (*n* = 28)	14	1.0		1.0	
High infiltration of tumour or stromal CD8^+^ IC (*n* = 14)	7	0.46 (0.047–4.6)		0.32 (0.028–3.8)	
High infiltration of tumour and stromal CD8^+^ IC (*n* = 24)	42	4.3 (1.1–16.3)		3.5 (0.78–15.9)	
*Combined stromal infiltration of PD-1*^*+*^ *and CD8*^*+*^ *IC*			**0.027**		
Low infiltration of stromal PD-1^+^ and CD8^+^ IC (*n* = 28)	11	1.0			
High infiltration of stromal PD-1^+^ or CD8^+^ IC (*n* = 17)	18	1.8 (0.32–10.1)			
High infiltration of stromal PD-1^+^ and CD8^+^ IC (*n* = 20)	45	6.8 (1.5–30.2)			
*Combined stromal infiltration of PD-L1*^*+*^ *and CD8*^*+*^ *IC*			**0.024**		
Low infiltration of stromal PD-L1^+^ and CD8^+^ IC (*n* = 30)	7	1.0			
High infiltration of stromal PD-L1^+^ or CD8^+^ IC (*n* = 16)	44	10.9 (1.9–62.1)			
High infiltration of PD-L1^+^ and CD8^+^ IC (*n* = 18)	33	7.0 (1.2–39.8)			

Statistically significant *p* < 0.05 values are in bold

The levels of PD-L1^+^, PD-1^+^, CD8^+^, CD3^+^ and CD4^+^ immune cell infiltration in tumour and/or stroma in samples of both cohorts are presented in Supplementary Table 3. No significant differences were observed between both groups. Univariate analysis revealed a significant association between stromal PD-1^+^ immune cell infiltration, stromal CD3^+^ immune cell infiltration, stromal CD4^+^ immune cell infiltration, combined tumour and stromal infiltration of CD8^+^ immune cells, combined intermediate-to-high stromal infiltration of CD8^+^ and PD-1^+^ immune cells and combined stromal infiltration by PD-L1^+^ and CD8^+^ immune cells and DCB (Table 2). However, multivariate analysis could not confirm these associations (Table 2), and the univariate associations were not confirmed by univariate analysis in the validation cohort (Supplementary Table 4).

**Fig. 2:**
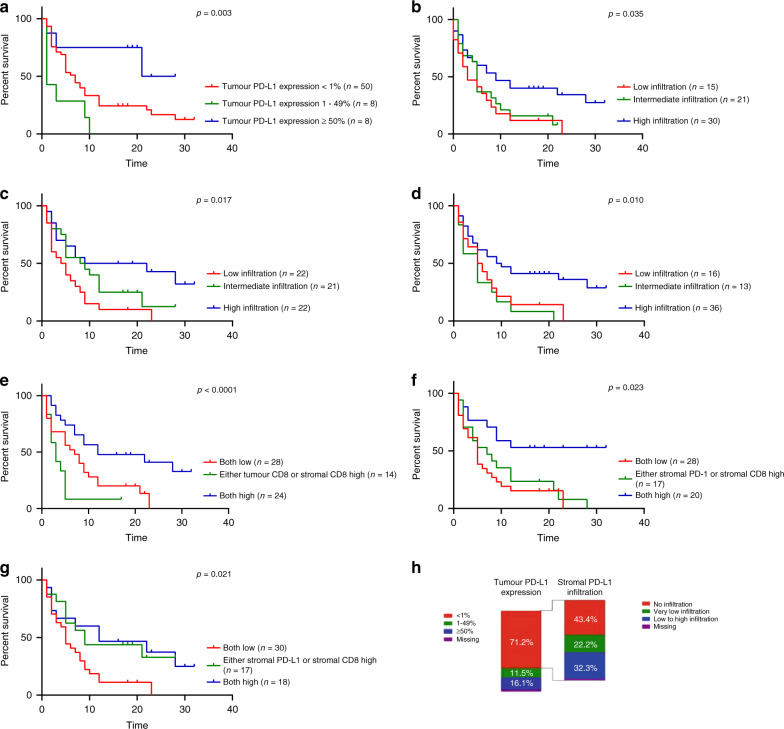
Kaplan–Meier plots for the derivation cohort. **a** Survival of tumour PD-L1 expression in the derivation cohort. **b** Survival of stromal CD8^+^ infiltration in the derivation cohort. **c** Survival of stromal CD3^+^ infiltration in the derivation cohort. **d** Survival of stromal CD4^+^ infiltration in the derivation cohort. **e** Survival of combined tumour and stromal CD8^+^ infiltration in the derivation cohort. **f** Survival of stromal combined PD-1^+^ and CD8^+^ infiltration in the derivation cohort. **g** Survival of stromal combined PD-L1^+^ and CD8^+^ infiltration in the derivation cohort. **h** Distribution of stromal PD-L1 expression in tumour PD-L1-negative patients (tumour PD-L1 expression < 1%).

Overall survival in all categories is shown in Table 3 for the derivation and the validation cohorts. In the derivation cohort, high tumour PD-L1 expression (*p* = 0.003), high infiltration of stromal CD8^+^ immune cells (*p* = 0.035), high infiltration of stromal CD3^+^ immune cells (*p* = 0.017), high infiltration of stromal CD4^+^ immune cells (*p* = 0.010), both high infiltration of stromal and tumour CD8^+^ immune cells (*p* < 0.0001), both high infiltration of stromal PD-1^+^ and CD8^+^ immune cells (*p* = 0.023) and both high infiltration of stromal PD-L1^+^ and CD8^+^ immune cells (*p* = 0.021) were associated with longer OS (Fig. 2a–g, Table 3).

**Table 3:** Overall survival of PD-L1, PD-1, CD8, CD4 and CD3 is shown for the derivation and validation cohorts.

Derivation cohort			Validation cohort		
Variables	Median OS (months)	*p* value	Variables	Median OS (months)	*p* value
*Tumour PD-L1 expression*		**0.003**	*Tumour PD-L1 expression*		0.73
<1% (*n* = 50)	5.0		<1% (*n* = 49)	6.0	
1–49% (*n* = 8)	1.0		1–49% (*n* = 8)	6.0	
≥50% (*n* = 8)	21.0		≥50% (*n* = 13)	8.0	
*Stromal infiltration of PD-L1*^*+*^ *IC*		0.68	*Stromal infiltration of PD-L1*^*+*^ *IC*		0.72
Low infiltration (*n* = 40)	5.0		Low infiltration (*n* = 44)	6.0	
Intermediate infiltration (*n* = 15)	7.0		Intermediate infiltration (*n* = 14)	9.0	
High infiltration (*n* = 9)	5.0		High infiltration (*n* = 10)	9.0	
*Stromal infiltration of PD-1*^*+*^ *IC*		0.080	*Stromal infiltration of PD-1*^*+*^ *IC*		0.13
Low infiltration (*n* = 38)	5.0		Low infiltration (*n* = 43)	6.0	
Intermediate infiltration (*n* = 21)	9.0		Intermediate infiltration (*n* = 13)	6.0	
High infiltration (*n* = 7)	NR		High infiltration (*n* = 14)	12.0	
*Tumour infiltration of CD8*^*+*^ *IC*		0.062	*Tumour infiltration of CD8*^*+*^ *IC*		**0.024**
Low infiltration (*n* = 35)	5.0		Low infiltration (*n* = 41)	5.0	
Intermediate infiltration (*n* = 18)	7.0		Intermediate infiltration (*n* = 9)	6.0	
High infiltration (*n* = 14)	5.0		High infiltration (*n* = 20)	12.0	
*Stromal infiltration of CD8*^*+*^ *IC*		**0.035**	*Stromal infiltration of CD8*^*+*^ *IC*		**0.007**
Low infiltration (*n* = 15)	5.0		Low infiltration (*n* = 14)	6.0	
Intermediate infiltration (*n* = 21)	5.0		Intermediate infiltration (*n* = 24)	4.0	
High infiltration (*n* = 30)	9.0		High infiltration (*n* = 32)	11.0	
*Stromal infiltration of CD3*^*+*^ *IC*		**0.017**	*Stromal infiltration of CD3*^*+*^ *IC*		0.16
Low infiltration (*n* = 22)	3.0		Low infiltration (*n* = 27)	5.0	
Intermediate infiltration (*n* = 21)	7.0		Intermediate infiltration (*n* = 28)	8.0	
High infiltration (*n* = 22)	8.0		High infiltration (*n* = 15)	10.0	
*Stromal infiltration of CD4*^*+*^ *IC*		**0.010**	*Stromal infiltration of CD4*^*+*^ *IC*		**0.018**
Low infiltration (*n* = 16)	5.0		Low infiltration (*n* = 15)	6.0	
Intermediate infiltration (*n* = 13)	5.0		Intermediate infiltration (*n* = 23)	3.0	
High infiltration (*n* = 36)	9.0		High infiltration (*n* = 32)	10.0	
*Combined tumour and stromal infiltration of CD8*^*+*^ *IC*		**<0.0001**	*Combined tumour and stromal infiltration of CD8*^*+*^ *IC*		**0.008**
Low infiltration of tumour and stromal CD8^+^ IC (*n* = 28)	5.0		Low infiltration of tumour and stromal CD8^+^ IC (*n* = 31)	5.0	
High infiltration of tumour or stromal CD8^+^ IC (*n* = 14)	2.0		High infiltration of tumour or stromal CD8^+^ IC (*n* = 17)	6.0	
High infiltration of tumour and stromal CD8^+^ IC (*n* = 24)	12.0		High infiltration of tumour and stromal CD8^+^ IC (*n* = 22)	12.0	
*Combined stromal infiltration of PD-1*^*+*^ *and CD8*^*+*^ *IC*		**0.023**	*Combined stromal infiltration of PD-1*^*+*^ *and CD8*^*+*^ *IC*		**0.027**
Low infiltration of stromal PD-1^+^ and CD8^+^ IC (*n* = 28)	5.0		Low infiltration of stromal PD-1^+^ and CD8^+^ IC (*n* = 33)	6.0	
High infiltration of stromal PD-1^+^ or CD8^+^ IC (*n* = 17)	7.0		High infiltration of stromal PD-1^+^ or CD8^+^ IC (*n* = 15)	3.0	
High infiltration of stromal PD-1^+^ and CD8^+^ IC (*n* = 20)	9.0		High infiltration of stromal PD-1^+^ and CD8^+^ IC (*n* = 22)	12.0	
*Combined stromal infiltration of PD-L1*^*+*^ *and CD8*^*+*^ *IC*		**0.021**	*Combined stromal infiltration of PD-L1*^*+*^ *and CD8*^*+*^ *IC*		0.18
Low infiltration of stromal PD-L1^+^ and CD8^+^ IC (*n* = 30)	5.0		Low infiltration of stromal PD-L1^+^ and CD8^+^ IC (*n* = 31)	5.0	
High infiltration of stromal PD-L1^+^ or CD8^+^ IC (*n* = 16)	9.0		High infiltration of stromal PD-L1^+^ or CD8^+^ IC (*n* = 19)	6.0	
High infiltration of stromal PD-L1^+^ and CD8^+^ IC (*n* = 18)	7.0		High infiltration of stromal PD-L1^+^ and CD8^+^ IC (*n* = 18)	12.0	

Statistically significant *p* < 0.05 values are in bold

In the validation cohort, longer OS was observed for patients with high infiltration of CD8^+^ immune cells, (*p* = 0.024), patients with high expression of stromal CD8^+^ immune cells (*p* = 0.007), patients with high infiltration of stromal and tumour-infiltrating CD8^+^ immune cells (*p* = 0.008) and patients with high stromal infiltration of both PD-1^+^ and CD8^+^ immune cells (*p* = 0.027, Supplementary Fig. 1; Table 3).

### In the total cohort, combined tumour and stromal CD8^+^ immune cell infiltration is associated with DCB in multivariate analysis, and immune cell markers are associated with OS

As we were not able to confirm our results of the derivation cohort in the validation cohort for DCB, we decided to combine both cohorts to enlarge the cohort size. In the total cohort, approximately 15% of all patients had positive membranous tumour PD-L1 staining of ≥50%, and 71.2% of all patients had less than 1% membranous tumour PD-L1 staining (Supplementary Table 5; Fig. 2h). In these tumour PD-L1-negative tumours, more than 50% of the patients had stromal infiltration of PD-L1-positive immune cells (Fig. 2h).

In the total cohort, univariate analyses showed that, besides the previously described tumour PD-L1 expression, also stromal CD3^+^, stromal CD4^+^, combined stromal infiltration of CD8^+^ and PD-1^+^ immune cells and tumour and stromal infiltration of CD8^+^ immune cells were associated with DCB (Supplementary Table 6). Multivariate analysis showed that infiltration of combined stromal and tumour CD8^+^ immune cells was associated with DCB (Supplementary Table 6).

Median OS was significantly longer for patients with high stromal PD-1^+^ immune cell infiltration (*p* = 0.015), patients with high tumour CD8^+^ immune cell infiltration (*p* = 0.004), patients with high stromal CD8^+^ immune cell infiltration (*p* = 0.001), patients with high infiltration of stromal CD3^+^ immune cells (*p* = 0.004), patients with high stromal CD4^+^ immune cell infiltration (*p* < 0.0001), patients with high infiltration of both tumour and stromal CD8^+^ immune cell infiltration (*p* < 0.0001), patients with high stromal infiltration of both PD-1^+^ and CD8^+^ immune cells (*p* = 0.001) and patients with high infiltration of both stromal PD-L1^+^ and CD8^+^ immune cells (*p* = 0.013) (Supplementary Fig. 2a–h, Supplementary Table 7).

### Tumour PD-L1 expression and immune cell infiltration markers predict DCB when analysed on tumour tissue taken after the last line of therapy, but not on archival samples

To address the influence of tumour biopsy timing (before or after the last line of systemic treatment), we performed a post hoc analysis between those patient groups. Baseline characteristics were equal with the exception of ECOG performance score (*p* = 0.002) (Supplementary Table 8). Infiltration levels of immune cells were equal (Supplementary Table 9). In the ACT biopsy group, tumour PD-L1 expression associated with DCB rate (DCB rate 69% for PD-L1 ≥ 50%, OR 7.4, 95% CI 1.9–28.6), but not in the BCT biopsy group (Table 4). In univariate analyses, stromal PD-1^+^ immune cell infiltration, tumour CD8^+^ immune cell infiltration, stromal CD8^+^ immune cell infiltration, stromal CD3^+^ immune cell infiltration, combined tumour and stromal CD8^+^ immune cell infiltration, combined stromal infiltration of PD-1^+^ and CD8^+^ immune cells and combined stromal PD-L1^+^ and CD8^+^ immune cell infiltration were associated with DCB in the ACT biopsy group. In the BCT biopsy group, no significant associations were found. Multivariate analysis showed that tumour PD-L1 and combined tumour and stromal infiltration of CD8^+^ immune cells were associated with DCB in the ACT biopsy group (Table 5).

**Table 4:** Results of univariate analysis of PD-L1, PD-1, CD8, CD4 and CD3 in relation to durable clinical benefit (DCB) are shown for biopsies taken before chemotherapy (BCT) and biopsies taken after the last line of chemotherapy (ACT).

BCT biopsies				ACT biopsies			
	DCB rates (%)	Univariate OR (95% CI)	*p* value		DCB rates (%)	Univariate OR (95% CI)	*p* value
*Tumour PD-L1 expression*			0.88	*Tumour PD-L1 expression*			**0.013**
<1% (*n* = 52)	21	1		<1% (*n* = 47)	23	1	
1–49% (*n* = 7)	14	0.62 (0.068–5.7)		1–49% (*n* = 9)	22	0.94 (0.17–5.2)	
≥50% (*n* = 8)	25	1.2 (0.22–7.0)		≥50% (*n* = 13)	69	7.4 (1.9–28.6)	
*Stromal infiltration of PD-L1*^*+*^ *IC*			0.60	Stromal infiltration of PD-L1^**+**^ IC			0.22
Low infiltration (*n* = 39)	26	1		Low infiltration (*n* = 45)	24	1	
Intermediate infiltration (*n* = 15)	13	0.45 (0.085–2.3)		Intermediate infiltration (*n* = 14)	43	2.3 (0.66–8.2)	
High infiltration (*n* = 11)	18	0.64 (0.12–3.5)		High infiltration (*n* = 8)	50	3.1 (0.66–14.5)	
*Stromal infiltration of PD-1*^*+*^ *IC*			0.55	Stromal infiltration of PD-1^**+**^ IC			**0.006**
Low infiltration (*n* = 40)	25	1		Low infiltration (*n* = 41)	17	1	
Intermediate infiltration (*n* = 17)	12	0.40 (0.078–2.1)		Intermediate infiltration (*n* = 17)	41	3.4 (0.96–12.0)	
High infiltration (*n* = 9)	22	0.86 (0.15–4.8)		High infiltration (*n* = 12)	67	9.7 (2.3–41.4)	
*Tumour infiltration of CD8*^*+*^ *IC*			0.52	Tumour infiltration of CD8^**+**^ IC			**0.009**
Low infiltration (*n* = 37)	22	1		Low infiltration (*n* = 39)	18	1	
Intermediate infiltration (*n* = 11)	9	0.36 (0.040–3.3)		Intermediate infiltration (*n* = 16)	31	2.1 (0.55–7.9)	
High infiltration (*n* = 18)	28	1.4 (0.38–5.1)		High infiltration (*n* = 16)	63	7.6 (2.1–28.0)	
*Stromal infiltration of CD8*^*+*^ *IC*			0.74	Stromal infiltration of CD8^**+**^ IC			**0.044**
Low infiltration (*n* = 11)	18	1		Low infiltration (*n* = 18)	17	1	
Intermediate infiltration (*n* = 28)	18	0.98 (0.16–6.0)		Intermediate infiltration (*n* = 17)	18	1.1 (0.19–6.2)	
High infiltration (*n* = 27)	26	1.6 (0.27–9.1)		High infiltration (*n* = 35)	46	4.2 (1.0–17.2)	
*Stromal infiltration of CD3*^*+*^ *IC*			0.20	Stromal infiltration of CD3^**+**^ IC			**0.035**
Low infiltration (*n* = 23)	9	1		Low infiltration (*n* = 26)	19	1	
Intermediate infiltration (*n* = 26)	31	4.7 (0.88–24.9)		Intermediate infiltration (*n* = 23)	26	1,5 (0.39–5.7)	
High infiltration (*n* = 17)	24	3.2 (0.52–20.2)		High infiltration (*n* = 20)	55	5.1 (1.4–19.1)	
*Stromal infiltration of CD4*^*+*^ *IC*			0.31	Stromal infiltration of CD4^**+**^ IC			0.10
Low infiltration (*n* = 16)	19	1		Low infiltration (*n* = 15)	20	1	
Intermediate infiltration (*n* = 19)	11	0.51 (0.074–3.5)		Intermediate infiltration (*n* = 17)	18	0.86 (0.15–5.1)	
High infiltration (*n* = 31)	29	1.8 (0.41–7.8)		High infiltration (*n* = 37)	43	3.0 (0.74–12.6)	
*Combined tumour and stromal infiltration of CD8*^*+*^ *IC*			0.64	Combined tumour and stromal infiltration of CD8^**+**^ IC			**0.008**
Low infiltration of tumour and stromal CD8^+^ IC (*n* = 28)	21	1		Low infiltration of tumour and stromal CD8^+^ IC (*n* = 31)	19	1	
High infiltration of tumour or stromal CD8^+^ IC (*n* = 20)	15	0.65 (0.14–3.0)		High infiltration of tumour or stromal CD8^+^ IC (*n* = 11)	9	0.42 (0.044–3.9)	
High infiltration of tumour and stromal CD8^+^ IC (*n* = 18)	28	1.4 (0.36–5.6)		High infiltration of tumour and stromal CD8^+^ IC (*n* = 28)	54	4.8 (1.5–15–3)	
*Combined stromal infiltration of PD-1*^*+*^ *and CD8*^*+*^ *IC*			0.62	Combined stromal infiltration of PD-1^**+**^ and CD8^**+**^ IC			**0.001**
Low infiltration of stromal PD-1^+^ and CD8^+^ IC (*n* = 31)	19	1		Low infiltration of stromal PD-1^+^ and CD8^+^ IC (*n* = 30)	20	1	
High infiltration of stromal PD-1^+^ or CD8^+^ IC (*n* = 17)	29	1.7 (0.44–6.8)		High infiltration of stromal PD-1^+^ or CD8^+^ IC (*n* = 15)	7	0.29 (0.031–2.6)	
High infiltration of stromal PD-1^+^ and CD8^+^ IC (*n* = 18)	17	0.83 (0.18–3.8)		High infiltration of stromal PD-1^+^ and CD8^+^ IC (*n* = 24)	63	6.7 (2.0–22.5)	
*Combined stromal infiltration of PD-L1*^*+*^ *and CD8*^*+*^ *IC*			0.44	*Combined stromal infiltration of PD-L1*^*+*^ *and CD8*^*+*^ *IC*			**0.043**
Low infiltration of stromal PD-L1^+^ and CD8^+^ IC (*n* = 31)	19	1		Low infiltration of stromal PD-L1^+^ and CD8^+^ IC (*n* = 30)	17	1	
High infiltration of stromal PD-L1^+^ or CD8^+^ IC (*n* = 15)	33	2.1 (0.52–8.4)		High infiltration of stromal PD-L1^+^ or CD8^+^ IC (*n* = 20)	35	2.7 (0.71–10.2)	
High infiltration of stromal PD-L1^+^ and CD8^+^ IC (*n* = 19)	16	0.78 (0.17–3.6)		High infiltration of stromal PD-L1^+^ and CD8^+^ IC (*n* = 17)	53	5.6 (1.5–21.8)	

Statistically significant *p* < 0.05 values are in bold

**Table 5:** Multivariate analysis for biopsies taken after the last line of therapy (ACT).

	DCB rates (%)	Multivariate OR (95% CI)	*p* value
*Tumour PD-L1 expression*			**0.047**
<1% (*n* = 47)	23	1.0	
1–50% (*n* = 9)	22	0.78 (0.13–4.8)	
≥50% (*n* = 13)	69	6.5 (1.4–30.7)	
*Combined tumour and stromal infiltration of* *CD8*^*+*^* IC*			**0.012**
Low infiltration of tumour and stromal CD8^+^ IC (*n* = 31)	19	1.0	
High infiltration of tumour or stromal CD8^+^ IC (*n* = 11)	9	0.29 (0.026–3.1)	
High infiltration of tumour and stromal CD8^+^ IC (*n* = 28)	54	4.5 (1.3–15.9)	

Statistically significant *p* < 0.05 values are in bold

In the ACT biopsy group, we found that high infiltration of stromal PD-1^+^ immune cell infiltration (*p* = 0.005), high infiltration of tumour CD8^+^ immune cells (*p* = 0.019), high infiltration of stromal CD8^+^ immune cells (*p* = 0.001), high infiltration of CD3^+^ immune cells (*p* = 0.003), high infiltration of CD4^+^ immune cells (*p* = 0.001), both high infiltration of tumour and stromal CD8^+^ immune cells (*p* < 0.0001), high infiltration of both PD-1^+^ and CD8^+^ immune cells (*p* < 0.0001) and both high infiltration of stromal PD-L1^+^ and CD8^+^ immune cells (*p* = 0.002) were associated with longer OS (Supplementary Fig. 3a–h, Table 6). In the BCT biopsy group, we found no associations between OS and immune cell infiltration.

**Table 6:** Overall survival of PD-L1, PD-1, CD8, CD4 and CD3 is shown for biopsies taken before chemotherapy (BCT) and biopsies taken after the last line of chemotherapy (ACT).

BCT biopsies			ACT biopsies		
	Median OS (months)	*p* value		Median OS (months)	*p* value
*Tumour PD-L1 expression*		0.67	*Tumour PD-L1 expression*		0.11
<1% (*n* = 52)	6.0		<1% (*n* = 47)	6.0	
1–49% (*n* = 7)	1.0		1–49% (*n* = 9)	6.0	
≥50% (*n* = 8)	3.0		≥50% (*n* = 13)	18.0	
*Stromal infiltration of PD-L1*^*+*^ *IC*		0.81	Stromal infiltration of PD-L1^**+**^ IC		0.10
Low infiltration (*n* = 39)	6.0		Low infiltration (*n* = 45)	6.0	
Intermediate infiltration (*n* = 15)	6.0		Intermediate infiltration (*n* = 14)	18.0	
High infiltration (*n* = 11)	5.0		High infiltration (*n* = 8)	12.0	
*Stromal infiltration of PD-1*^*+*^ *IC*		0.38	Stromal infiltration of PD-1^**+**^ IC		**0.005**
Low infiltration (*n* = 40)	5.0		Low infiltration (*n* = 41)	5.0	
Intermediate infiltration (*n* = 17)	4.0		Intermediate infiltration (*n* = 17)	11.0	
High infiltration (*n* = 9)	11.0		High infiltration (*n* = 12)	NR	
*Tumour infiltration of CD8*^*+*^ *IC*		0.39	Tumour infiltration of CD8^**+**^ IC		**<0.0001**
Low infiltration (*n* = 37)	6.0		Low infiltration (*n* = 39)	4.0	
Intermediate infiltration (*n* = 11)	5.0		Intermediate infiltration (*n* = 16)	8.0	
High infiltration (*n* = 18)	5.0		High infiltration (*n* = 16)	NR	
*Stromal infiltration of CD8*^*+*^ *IC*		0.26	Stromal infiltration of CD8^**+**^ IC		**0.001**
Low infiltration (*n* = 11)	6.0		Low infiltration (*n* = 18)	3.0	
Intermediate infiltration (*n* = 28)	5.0		Intermediate infiltration (*n* = 17)	5.0	
High infiltration (*n* = 27)	6.0		High infiltration (*n* = 35)	14.0	
*Stromal infiltration of CD3*^*+*^ *IC*		0.22	Stromal infiltration of CD3^**+**^ IC		**0.003**
Low infiltration (*n* = 23)	5.0		Low infiltration (*n* = 26)	3.0	
Intermediate infiltration (*n* = 26)	6.0		Intermediate infiltration (*n* = 23)	9.0	
High infiltration (*n* = 17)	5.0		High infiltration (*n* = 20)	23.0	
*Stromal infiltration of CD4*^*+*^ *IC*		0.2	Stromal infiltration of CD4^**+**^ IC		**0.001**
Low infiltration (*n* = 16)	6.0		Low infiltration (*n* = 15)	5.0	
Intermediate infiltration (*n* = 19)	4.0		Intermediate infiltration (*n* = 17)	2.0	
High infiltration (*n* = 31)	6.0		High infiltration (*n* = 37)	14.0	
*Combined tumour and stromal infiltration of CD8*^*+*^ *IC*		0.12	Combined tumour and stromal infiltration of CD8^**+**^ IC		**<0.0001**
Low infiltration of tumour and stromal CD8^+^ IC (*n* = 28)	6.0		Low infiltration of tumour and stromal CD8^+^ IC (*n* = 31)	5.0	
High infiltration of tumour or stromal CD8^+^ IC (*n* = 20)	4.0		High infiltration of tumour or stromal CD8^+^ IC (*n* = 11)	3.0	
High infiltration of tumour and stromal CD8^+^ IC (*n* = 18)	6.0		High infiltration of tumour and stromal CD8^+^ IC (*n* = 28)	23.0	
*Combined stromal infiltration of PD-1*^*+*^ *and CD8*^*+*^ *IC*		0.72	Combined stromal infiltration of PD-1^**+**^ and CD8^**+**^ IC		**<0.0001**
Low infiltration of stromal PD-1^+^ and CD8^+^ IC (*n* = 31)	5.0		Low infiltration of stromal PD-1^+^ and CD8^+^ IC (*n* = 30)	5.0	
High infiltration of stromal PD-1^+^ or CD8^+^ IC (*n* = 17)	4.0		High infiltration of stromal PD-1^+^ or CD8^+^ IC (*n* = 15)	5.0	
High infiltration of stromal PD-1^+^ and CD8^+^ IC (*n* = 18)	6.0		High infiltration of stromal PD-1^+^ and CD8^+^ IC (*n* = 24)	23.0	
*Combined stromal infiltration of PD-L1*^*+*^ *and CD8*^*+*^ *IC*		0.83	*Combined stromal infiltration of PD-1*^*+*^ *and CD8*^*+*^ *IC*		**0.002**
Low infiltration of stromal PD-L1^+^ and CD8^+^ IC (*n* = 31)	6.0		Low infiltration of stromal PD-L1^+^ and CD8^+^ IC (*n* = 30)	3.0	
High infiltration of stromal PD-L1^+^ or CD8^+^ IC (*n* = 15)	5.0		High infiltration of stromal PD-L1^+^ or CD8^+^ IC (*n* = 20)	8.0	
High infiltration of stromal PD-L1^+^ and CD8^+^ IC (*n* = 19)	6.0		High infiltration of stromal PD-L1^+^ and CD8^+^ IC (*n* = 17)	23.0	

Statistically significant *p* < 0.05 values are in bold

## Discussion

In this explorative study, we found that tumour PD-L1 expression was predictive for DCB in the derivation cohort, and in patients who have had a biopsy after the last line of therapy. Although only confirmed in the latter group and in the total cohort, the infiltration of both tumour and stromal CD8^+^ immune cells seems to play an additional role in the predictive value for DCB. High infiltration of stromal CD8^+^ immune cells, high infiltration of stromal CD4^+^ immune cells, high infiltration of both tumour and stromal CD8^+^ immune cells and high infiltration of PD-1^+^ and CD8^+^ immune cells were associated with longer OS in all subsets. This implies that the microenvironment of the tumour and the stromal compartment is important for obtaining a durable response to nivolumab.

Consistent with earlier studies in melanoma and NSCLC patients, tumour and stromal infiltration of CD8^+^ immune cells were associated with DCB.^14,22,23^ In line with these results, we also observed stromal CD8^+^ infiltration alone to be associated with OS. In contrast to a small study among NSCLC patients treated with nivolumab, that showed that low PD-1 to CD8 ratio was associated with DCB and progression-free survival (PFS), we found that high stromal infiltration of both PD-1^+^ and CD8^+^ immune cells was associated with DCB and OS. Similar findings were reported in previous studies in melanoma patients.^14,24^ The upregulation of PD-1 on CD8^+^ T cells under chronic and persistent antigen stimulation is well described.^2^ Blocking this PD-1 receptor with anti-PD-1 therapy could therefore result in activation of CD8^+^ T cells, and as a result enhance tumour regression.

The role of CD4^+^ T cells is increasingly being studied in the T-cell response against tumours. CD4^+^ T cells are responsible for cytokine production and T-cell regulation. A previous meta-analysis described that stromal infiltration of CD4^+^ T cells is associated with better OS and disease-specific survival.^25^ In our study, the presence of stromal CD4^+^ T cells was also associated with a longer OS to nivolumab in the second-line setting.

Importantly, this study showed differences for the predictive value of IHC markers between biopsies obtained before and after the line of systemic treatment. While tumour PD-L1 and combined tumour and stromal infiltration of CD8^+^ immune cells were associated with DCB and OS in the ACT biopsy group, there were no predictive markers for DCB and OS in the BCT biopsy group. Increasing evidence supports the hypothesis that the anticancer effects of chemotherapy not only cause cancer apoptosis, but also modulate the immune system. The widely used drug cisplatin, for example, induces upregulation of MHC class I expression on tumour cells and antigen-presenting cells, improves the proliferation of immune effector cells and can downregulate immunosuppressive components in the tumour microenvironment.^26^ Changes in PD-L1 expression and immune contexture during anticancer treatment have been reported.^27–30^ OS benefit of pembrolizumab over docetaxel has been demonstrated for both ≥50% and ≥1% tumour PD-L1 expression cohorts, regardless of the time the samples were collected,^31^ whereas in this study, no association between tumour PD-L1 expression and stromal infiltrates and response was found in the BCT biopsy group. Our results suggest that obtaining a new biopsy before the start of treatment is warranted in the case the patient received chemotherapy.

Recently, an increasing number of studies are indicating that tumour-infiltrating immune cells like dendritic cells and macrophages contribute to the antitumour effect of anti-PD-(L)1 therapy.^32,33^ In this study, we observed that patients with high stromal infiltration of PD-L1^+^ and CD8^+^ immune cells had a better OS than patients with low infiltration, although we could not confirm these results in our validation cohort. We did not selectively stain for dendritic cells or macrophages, but our results suggest that stromal expression of PD-L1 prolongs OS.

The use of PD-L1 expression as a biomarker for immune checkpoint inhibitors has been debated. In previous trials with nivolumab, the association between PD-L1 IHC and outcome parameters in terms of the overall response rate (ORR), OS and PFS has been conflicting.^4,5,34^ In our study, we found that tumour PD-L1 expression was associated with DCB, and with OS in only the derivation cohort. We also did not observe an association between stromal PD-L1 expression as a single marker and DCB. A previous study in patients treated with atezolizumab described durable responses in patients with PD-L1 positivity on immune cells alone.^35^ An explanation for this difference could be the difference in PD-L1 assays that were used to stain PD-L1, as this may explain misclassification of PD-L1 expression status.^11^

In this study, 71% of all patients had a negative tumour PD-L1 expression score. This contrasts with previous studies that reported prevalences between 17% and 22%.^4,5,9^ One possible explanation for this finding is the method used for quantifying tumour PD-L1 expression in this study. We used a broad scale of immune cell markers in addition to PD-L1 for staining biopsy samples, which resulted in a more comprehensive evaluation of the different cell types that expressed PD-L1. When visual assessment of the nuclei did not yield a clear distinction between malignant and immune cells, the use of dual CD4 and PD-L1 staining helped to make the distinction between malignant and immune cells, thereby preventing overestimation of PD-L1 scores. It cannot be excluded that macrophages expressing both PD-L1 and CD4 were counted as PD-L1-expressing tumour cells in a number of cases in previous studies, which may explain the difference between our observations and those reported in the literature, especially in the range of PD-L1 scoring below or just above the 1% range. Therefore, for future studies, simultaneous (multiplex) staining for PD-L1 and CD4 could result in improved accuracy of tumour PD-L1 assessments in biopsy samples.

There are several challenging aspects in the interpretation of our results. First, the heterogeneity and variability of PD-L1 expression within individual tumours may account for a limited external validity of our study, knowing that up to 35% of patients may be misclassified based on PD-L1 staining performed on small biopsy samples.^36–39^ Second, it is known that tumour PD-L1 expression levels can vary over time and be influenced by several host and environmental factors, such as TNM stage, chemotherapy and cytokines like interferon-α.^40–43^ In our study, both ACT and BCT biopsies were included. Post hoc analysis shows that ACT biopsies were more informative for response prediction than BCT biopsies. Randomisation of our cohort in a derivation and validation cohort could not confirm our results found in the total cohort, with the exception of the association of immune cell markers and OS. This might be explained by the small numbers of patients in both cohorts.

In summary, in this study, we observed that tumour PD-L1, stromal infiltration of CD3^+^ immune cells, combined tumour and stromal infiltration of CD8^+^ immune cells, combined stromal infiltration of PD-1^+^ and CD8^+^ immune cells and combined stromal infiltration of PD-L1^+^ and CD8^+^ immune cells are associated with DCB on nivolumab treatment. Although there were no major differences in baseline patient characteristics and the outcome to nivolumab treatment between the ACT and the BCT biopsy groups, we were able to identify several predictive markers in the ACT group, including tumour PD-L1 expression and combined tumour and stromal infiltration of CD8^+^ immune cells for DCB, and stromal PD-1^+^, CD8^+^, CD3^+^ and CD4^+^ immune cells and tumour CD8^+^ immune cells for OS. The same markers in BCT biopsies were not predictive for DCB. This indicates that the immune contexture may change during treatment with conventional chemotherapy and therefore, that obtaining a new biopsy before the start of nivolumab is preferable. Further research is needed to validate our findings in a larger cohort, and to examine whether the infiltration of both tumour and stromal CD8^+^ immune cells can be used as a biomarker during immune checkpoint inhibition in NSCLC patients.

## Supplementary information

Supplemental material and tables

Supplementary figures

## Data Availability

The data generated and analysed for the current investigation are not publicly available, but are available through the corresponding author on reasonable request.
