# Supplementary material for: Association of tumour and stroma PD-1, PD-L1, CD3, CD4 and CD8 expression with DCB and OS to nivolumab treatment in NSCLC patients pre-treated with chemotherapy
Source: Br J Cancer. 2020 May 20;123(3):392–402. doi: 10.1038/s41416-020-0888-5 (PMC7403301; doi:10.1038/s41416-020-0888-5)
Supplement: Supplementary file 1 — Supplemental material and tables [file 41416_2020_888_MOESM1_ESM.docx]

**Supplemental material**

**Supplementary methods**

*Immunohistochemistry (IHC) analysis*3-4 µm tissue slides were mounted on TOMO glass slides (Roche, Basel, Switzerland), 15 minutes baked at 60°C and brought to room temperature. Slides were stained for PD-L1, PD-1, CD8, CD3 and CD4 as listed in Table 1. All IHC steps were performed in Ventana Benchmark Ultra (Ventana, Tucson, USA) according to standard procedures from deparaffination up until hematoxylin counterstain. Antigen retrieval was performed using CC1 (pH8.5) for 24-32 minutes at 100°C. Antibodies were diluted with Ventana diluent, except for CD4 because this was done in Dako antibody diluent with reducing elements (catno. S3022) and incubated for 32-48 minutes. Detection and visualization was performed with Optiview DAB kit. Counterstaining was done with Hematoxylin II (Ventana, Tucson, USA). Slides were rinsed with EZ-Prep, luke warm soap, running tapwater, dehydrated with Ethanol 100%, cleared with Xylene and mounted with TissueTEK II film (Sakura).

PD-L1 28-8 PharmDx kit stainings were done according to company instructions with unchangeable staining protocol. Deparaffination/antigen retrieval was done with Low pH buffer for 20 minutes at 97°C. Other steps were done in the Dako Autolink 48 IHC staining device. Incubation with antibody was for 30 minutes. Rabbit linker was 30 minutes. Envision-HRP was 30 minutes, DAB was for 10 minutes and Enhancer was 5 minutes. Counterstaining was performed with hematoxylin. Slides were dehydrated with Ethanol 100%, cleared with Xylene and mounted with TissueTEK II film (Sakura).

**Supplementary Table 1.** Overview of antibodies used for immunohistochemical staining of patients tissue samples

| **Marker** | **Antibody used** | **Company** | **Catalog no.** | **Dilution** | **Incubation time** |
| --- | --- | --- | --- | --- | --- |
| PD-L1 | companion diagnostics kit antibody clone 28.8 | Dako North America, Inc. | - | prediluted of Dako | 30 min |
| PD1 | monoclonal mouse anti-human clone NAT105 | Cell Marque Corporation | 315M-94 | 1:100 | 32 min |
| CD8 | monoclonal rabbit anti-human clone SP16 | Spring Bioscience | M3160 | 1:50 | 32 min |
| CD3 | polyclonal rabbit anti-human | Dako North America Inc. | A0452 | 1:150 | 32 min |
| CD4 | monoclonal rabbit anti-human clone SP35 | Spring Bioscience | M3350 | 1:50 | 48 min |

**Supplementary Table 2.** Overview of categories scored and combined.

| Category | Categories scored | Categories combined |
| --- | --- | --- |
| Tumor PD-L1 expression | Tumor PD-L1 expression <1% | Tumor PD-L1 expression <1% |
|  | Tumor PD-L1 expression 1-5% | Tumor PD-L1 expression 1-49% |
|  | Tumor PD-L1 expression 5-10% |  |
|  | Tumor PD-L1 expression 10-25% |  |
|  | Tumor PD-L1 expression 25-49% |  |
|  | Tumor PD-L1 expression ≥50% | Tumor PD-L1 expression ≥50% |
| Tumor infiltration of CD8^+^ IC | No infiltration | Low infiltration |
|  | Very low infiltration | Intermediate infiltration |
|  | Low infiltration | High infiltration |
|  | Intermediate infiltration |  |
|  | High infiltration |  |
| Stromal infiltration of CD3^+^ IC | No infiltration | Low infiltration |
|  | Very low infiltration |  |
|  | Low infiltration |  |
|  | Intermediate infiltration | Intermediate infiltration |
|  | High infiltration | High infiltration |
| Stromal infiltration of CD4^+^ IC | No infiltration | Low infiltration |
|  | Very low infiltration |  |
|  | Low infiltration |  |
|  | Intermediate infiltration | Intermediate infiltration |
|  | High infiltration | High infiltration |
| Stromal infiltration of PD-L1^+^ IC | No infiltration | Low infiltration |
|  | Very low infiltration |  |
|  | Low infiltration | Intermediate infiltration |
|  | Intermediate infiltration | High infiltration |
|  | High infiltration |  |
| Stromal infiltration of CD8^+^ IC | No infiltration | Low infiltration |
|  | Very low infiltration |  |
|  | Low infiltration | Intermediate infiltration |
|  | Intermediate infiltration | High infiltration |
|  | High infiltration |  |
| Stromal infiltration of PD-1^+^ IC | No infiltration | Low infiltration |
|  | Very low infiltration |  |
|  | Low infiltration | Intermediate infiltration |
|  | Intermediate infiltration | High infiltration |
|  | High infiltration |  |

**Supplementary Table 3.** The distribution of PD-L1, CD8, CD4, CD3 scoring categories is shown for derivation and validation cohort.

|  | **Derivation cohort**  N (%) | **Validation cohort**  N (%) | ***p* value** |
| --- | --- | --- | --- |
| Tumor PD-L1 expression   - <1% - 1-49% - ≥50% | 50 (75.8)  8 (12.1)  8 (12.1) | 49 (70.0)  8 (11.4)  13 (18.6) | 0.58 |
| Stromal infiltration of PD-L1^+^ IC   - Low infiltration - Intermediate infiltration - High infiltration | 40 (62.5)  15 (23.4)  9 (14.1) | 44 (64.7)  14 (20.6)  10 (14.7) | 0.93 |
| Stromal infiltration of PD-1^+^ IC   - Low infiltration - Intermediate infiltration - High infiltration | 38 (57.6)  21 (31.8)  7 (10.6) | 43 (61.4)  13 (18.6)  14 (20.0) | 0.11 |
| Tumor infiltration of CD8^+^ IC   - Low infiltration - Intermediate infiltration - High infiltration | 35 (52.2)  18 (26.9)  14 (20.9) | 41 (58.6)  9 (12.9)  20 (28.6) | 0.11 |
| Stromal infiltration of CD8^+^ IC   - Low infiltration - Intermediate infiltration - High infiltration | 15 (22.7)  21 (31.8)  30 (45.5) | 14 (20.0)  24 (34.3)  32 (45.7) | 0.91 |
| Stromal infiltration of CD4^+^ IC   - Low infiltration - Intermediate infiltration - High infiltration | 16 (24.6)  13 (20.0)  36 (55.4) | 15 (21.4)  23 (32.9)  32 (45.7) | 0.24 |
| Stromal infiltration of CD3^+^ IC   - Low infiltration - Intermediate infiltration - High infiltration | 22 (33.8)  21 (32.3)  22 (33.8) | 27 (38.6)  28 (40.0)  15 (21.4) | 0.27 |

Abbreviations N number; IC immune cells.

**Supplementary Table 4.** Univariate analysis of PD-L1, CD8, CD4, CD3 for durable clinical benefit is

shown for the validation cohort.

|  | *Validation cohort* | | | |
| --- | --- | --- | --- | --- |
| Variables | **DCB rate %** | **Univariate OR (95%-CI)** | ***p* value** |  |
| Tumor PD-L1 expression   - <1% (n = 49) - 1-49% (n = 8) - ≥50% (n =13) | 27  38  39 | 1.0  1.7 (0.35-8.0)  1.7 (0.48-6.3) | 0.63 |  |
| Stromal infiltration of PD-L1^+^ IC   - Low infiltration (n = 44) - Intermediate infiltration (n = 14) - High infiltration (n = 10) | 32  22  30 | 1.0  0.58 (0.14-2.4)  0.92 (0.20-4.1) | 0.76 |  |
| Stromal infiltration of PD-1^+^ IC   - Low infiltration (n = 43) - Intermediate infiltration (n = 13) - High infiltration (n = 14) | 28  23  43 | 1.0  0.78 (0.18-3.3)  1.9 (0.56-6.8) | 0.48 |  |
| Tumor infiltration of CD8^+^ IC   - Low infiltration (n = 41) - Intermediate infiltration (n = 9) - High infiltration (n = 20) | 24  22  45 | 1.0  0.89 (0.16-5.0)  2.5 (0.82-7.9) | 0.23 |  |
| Stromal infiltration of CD8^+^ IC   - Low infiltration (n = 14) - Intermediate infiltration (n = 24) - High infiltration (n = 32) | 29  21  38 | 1.0  0.66 (0.14-3.0)  1.5 (0.38-5.9) | 0.41 |  |
| Stromal infiltration of CD3^+^ IC   - Low infiltration (n = 27) - Intermediate infiltration (n = 28) - High infiltration (n = 15) | 22  32  40 | 1.0  1.7 (0.50-5.5)  2.3 (0.59-9.2) | 0.47 |  |
| Stromal infiltration of CD4^+^ IC   - Low infiltration (n = 15) - Intermediate infiltration (n = 23) - High infiltration (n = 32) | 33  17  38 | 1.0  0.42 (0.092-1.9)  1.2 (0.33-4.4) | 0.28 |  |
| Combined tumor and stromal infiltration of CD8^+^ IC   - Low infiltration of tumor and stromal CD8^+^ IC (n = 31) - High infiltration of tumor CD8^+^ or stromal CD8^+^ IC (n = 17) - High infiltration of tumor and stromal CD8^+^ IC (n = 22) | 26  18  46 | 1.0  0.6 (0.14-2.7)  2.4 (0.75-7.7) | 0.15 |  |
| Combined stromal infiltration of PD-1^+^ and CD8^+^ IC   - Low infiltration of stromal PD-1^+^ and CD8^+^ IC (n = 33) - High infiltration of stromal PD-1^+^ or CD8^+^ IC (n = 15) - High infiltration of stromal PD-1^+^ and CD8^+^ IC (n = 22) | 27  20  41 | 1.0  0.67 (0.15-2.9)  1.8 (0.59-5.8) | 0.36 |  |
| Combined stromal infiltration of PD-L1^+^ and CD8^+^ IC   - Low infiltration of stromal PD-L1^+^ and CD8^+^ IC (n = 31) - High infiltration of stromal PD-L1^+^ or CD8^+^ IC (n = 19) - High infiltration of stromal PD-L1^+^ and CD8^+^ IC (n = 18) | 29  26  33 | 1.0  0.87 (0.24-3.1)  1.2 (0.35-4.3) | 0.90 |  |

Abbreviations DCB durable clinical benefit; OR odds ratio; IC immune cells.

**Supplementary table 5.** Clinicopathological characteristics. The distribution of PD-L1, CD8, CD4, CD3 scoring categories in the total cohort.

|  | **N (%)** |
| --- | --- |
| Tumor PD-L1 expression   - <1% - 1-49% - ≥50% - Missing | 99 (71.2)  16 (11.5)  21 (15.1)  3 (2.2) |
| Stromal infiltration of PD-L1^+^ IC   - Low infiltration - Intermediate infiltration - High infiltration - Missing | 84 (60.4)  29 (20.9)  19 (13.7)  7 (5.0) |
| Stromal infiltration of PD-1^+^ IC   - Low infiltration - Intermediate infiltration - High infiltration - Missing | 81 (58.3)  34 (24.5)  21 (15.1)  3 (2.2) |
| Tumor infiltration of CD8^+^ IC   - Low infiltration - Intermediate infiltration - High infiltration - Missing | 76 (54.7)  27 (19.4)  34 (24.5)  2 (1.4) |
| Stromal infiltration of CD8^+^ IC   - Low infiltration - Intermediate infiltration - High infiltration - Missing | 29 (20.9)  45 (32.4)  62 (44.6)  3 (2.2) |
| Stromal infiltration of CD4^+^ IC   - Low infiltration - Intermediate infiltration - High infiltration - Missing | 31 (22,3)  36 (25.9)  68 (48,9)  4 (2.9) |
| Stromal infiltration of CD3^+^ IC   - Low infiltration - Intermediate infiltration - High infiltration - Missing | 49 (35.3)  49 (35.3)  37 (26.6)  4 (2.9) |

Abbreviation N number.

**Supplementary Table 6.** Univariate and multivariate analysis of PD-L1, PD-1, CD8, CD4 and CD3 scoring categories is shown for the total cohort.

|  | *Total cohort* | | |  |  |
| --- | --- | --- | --- | --- | --- |
| Variables | **DCB rate (%)** | **Univariate OR (95%-CI)** | ***p* value** | **Multivariate OR (95%-CI)** | ***p* value** |
| Tumor PD-L1 expression   - <1% (n = 99) - 1-49% (n = 16) - ≥50% (n = 21) | 22  19  52 | 1.0  0.81 (0.21-3.1)  3.9 (1.4-10.2) | **0.019** | 1.0  0.72 (0.18-2.9)  2.7 (0.95-7.7) | 0.13 |
| Stromal infiltration of PD-L1^+^ IC   - Low infiltration (n = 84) - Intermediate infiltration (n = 29) - High infiltration (n = 19) | 25  28  32 | 1.0  1.1 (0.44-3.0)  1.4 (0.47-4.1) | 0.83 |  |  |
| Stromal infiltration of PD-1^+^ IC   - Low infiltration (n = 81) - Intermediate infiltration (n = 34) - High infiltration (n = 21) | 21  27  48 | 1.0  1.4 (0.53-3.4)  3.4 (1.2-9.4) | 0.057 |  |  |
| Tumor infiltration of CD8^+^ IC   - Low infiltration (n = 76) - Intermediate infiltration (n = 27) - High infiltration (n = 34) | 20  22  44 | 1.0  1.2 (0.40-3.4)  3.2 (1.3-7.8) | **0.028** |  |  |
| Stromal infiltration of CD8^+^ IC   - Low infiltration (n = 29) - Intermediate infiltration (n = 45) - High infiltration (n = 62) | 17  18  37 | 1.0  1.0 (0.30-3.6)  2.8 (0.95-8.4) | **0.041** |  |  |
| Stromal infiltration of CD3^+^ IC   - Low infiltration (n = 49) - Intermediate infiltration (n = 49) - High infiltration (n = 37) | 14  29  41 | 1.0  2.4 (0.8-6.6)  4.1 (1.5-11.5) | **0.028** |  |  |
| Stromal infiltration of CD4^+^ IC   - Low infiltration (n = 31) - Intermediate infiltration (n = 36) - High infiltration (n = 68) | 19  14  37 | 1.0  1.3 (0.12-13.2)  2.1 (0.23-18.3) | **0.030** |  |  |
| Combined tumor and stromal infiltration of CD8^+^ IC   - Low infiltration of tumor and stromal CD8^+^ IC (n = 59) - High infiltration of tumor CD8^+^ or stromal CD8^+^ IC (n = 31) - High infiltration of tumor and stromal CD8^+^ IC (n = 46) | 20  13  44 | 1.0  0.58 (0.17-2.0)  3.0 (1.3-7.1) | **0.006** | 1.0  0.55 (0.16-1.9)  2.7 (1.1-6.6) | **0.017** |
| Combined stromal infiltration of PD-1^+^ and CD8^+^ IC   - Low infiltration of stromal PD-1^+^ and CD8^+^ IC (n = 61) - High infiltration of stromal PD-1^+^ or CD8^+^ IC (n = 32) - High infiltration of PD-1^+^ and CD8^+^ IC(n = 42) | 20  19  43 | 1.0  0.94 (0.32-2.8)  3.1 (1.3-7.4) | **0.020** |  |  |
| Combined stromal PD-L1^+^ and CD8^+^ IC   - Low infiltration of stromal PD-L1^+^ and CD8^+^ IC (n = 61) - High infiltration of stromal PD-L1^+^ or CD8^+^ IC (n = 35) - High infiltration of stromal PD-L1^+^ and CD8^+^ IC (n = 36) | 18  34  33 | 1.0  2.4 (0.9-6.2)  2.3 (0.88-5.9) | 0.13 |  |  |

Abbreviations DCB durable clinical benefit; OR odds ratio; IC immune cells.

**Supplementary Table 7.** Overall Survival of PD-L1, PD-1, CD8, CD4 and CD3 scoring categories is shown in total cohort

| Total cohort | | |
| --- | --- | --- |
| **Variables** | **Median OS (months)** | ***p* value** |
| Tumor PD-L1 expression   - <1% (n = 99) - 1-49% (n = 16) - ≥50% (n = 21) | 6.0  1.0  13.0 | 0.16 |
| Stromal infiltration of PD-L1^+^ IC   - Low infiltration (n = 84) - Intermediate infiltration (n = 29) - High infiltration (n = 19) | 6.0  9.0  9.0 | 0.58 |
| Stromal infiltration of PD-1^+^ IC   - Low infiltration (n = 81) - Intermediate infiltration (n = 34) - High infiltration (n = 21) | 5.0  8.0  12.0 | **0.015** |
| Tumor infiltration of CD8^+^ IC   - Low infiltration (n = 76) - Intermediate infiltration (n = 27) - High infiltration (n = 34) | 5.0  6.0  11.0 | **0.004** |
| Stromal infiltration of CD8^+^ IC   - Low infiltration (n = 29) - Intermediate infiltration (n = 45) - High infiltration (n = 62) | 6.0  5.0  10.0 | **0.001** |
| Stromal infiltration of CD3^+^ IC   - Low infiltration (n = 49) - Intermediate infiltration (n = 49) - High infiltration (n = 37) | 5.0  8.0  9.0 | **0.004** |
| Stromal infiltration of CD4^+^ IC   - Low infiltration (n = 31) - Intermediate infiltration (n = 36) - High infiltration (n = 68) | 6.0  3.0  9.0 | **< 0.0001** |
| Combined tumor and stromal infiltration of CD8^+^ IC   - Low infiltration of tumor and stromal CD8^+^ IC (n = 59) - High infiltration of tumor CD8^+^ or stromal CD8^+^ IC (n = 31) - High infiltration of tumor CD8^+^ or stromal CD8^+^ IC (n = 46) | 5.0  4.0  12.0 | **< 0.0001** |
| Combined stromal infiltration of PD-1^+^ and CD8^+^ IC   - Low infiltration of stromal PD-1^+^ and CD8^+^ IC (n = 61) - High infiltration of stromal PD-1^+^ or CD8^+^ IC (n = 32) - High infiltration of stromal PD-1^+^ and CD8^+^ IC (n = 42) | 5.0  4.0  12.0 | **0.001** |
| Combined stromal infiltration of PD-L1^+^ and CD8^+^ IC   - Low infiltration of stromal PD-L1^+^ and CD8^+^ IC (n = 61) - High infiltration of stromal PD-L1^+^ or CD8^+^ IC (n = 35) - High infiltration of stromal PD-L1^+^ and CD8^+^ IC (n = 36) | 5.0  7.0  12.0 | **0.013** |

Abbreviations OS Overall Survival; IC immune cells.

**Supplementary Table 8.** Baseline characteristics are shown for the ACT and BCT groups.

|  | **BCT**  N (%) | **ACT**  N (%) | ***p* value** |
| --- | --- | --- | --- |
| Number of patients Mean age in years, mean ± SD | 68  61.6±8.9 | 71 64.3±9.1 | 0.084 |
| Sex  Male   Female | 37 (54.4) 31 (45.6) | 40 (56.3) 31 (43.7) | 0.87 |
| Histological subtypes  Squamous  Non-squamous  Other (adenosquamous, LCNEC, NOS, unknown) | 23 (33.8)  44 (64.7) 1 (1.5) | 18 (25.4) 50 (70.4) 3 (4.2) | 0.51 |
| ECOG Performance Score  0  1  2 or >  Unknown | 1 (1.5)  46 (67.6)  20 (29.4)  1 (1.5) | 14 (19.7)  38 (53.5) 18 (25.4) 1 (1.4) | **0.002** |
| DCB rate  Patients with DCB  Patients without DCB | 14 (20.6) 54 (79.4) | 22 (31.0)  49 (69.0) | 0.18 |
| Treatment duration  0-3 months  > 3 months | 45  23 | 45  26 | 0.73 |

Abbreviations BCT biopsy taken before chemotherapy; ACT biopsy taken after last line of chemotherapy; N numbers; LCNEC Large cell neuroendocrine carcinoma; NOS not otherwise specified; ECOG Eastern Cooperative Oncology Group; DCB durable clinical benefit.

**Supplementary Table 9.** Infiltration levels stratified for BCT cohort and ACT cohort.

|  | **BCT cohort**  N (%) | **ACT cohort**  N (%) | ***p* value** |
| --- | --- | --- | --- |
| Tumor PD-L1 expression   - <1% - 1-49% - ≥50% | 52 (77.6)  7 (10.4)  8 (11.9) | 47 (68.1)  9 (13.0)  13 (18.8) | 0.44 |
| Stromal infiltration PD-L1^+^ IC   - Low infiltration - Intermediate infiltration - High infiltration | 39 (60.0)  15 (23.1)  11 (16.9) | 45 (67.2)  14 (20.9)  8 (11.9) | 0.64 |
| Stromal infiltration of PD-1^+^ IC   - Low infiltration - Intermediate infiltration - High infiltration | 40 (60.6)  17 (25.8)  9 (13.6) | 41 (58.6)  17 (24.3)  12 (17.1) | 0.85 |
| Tumor infiltration of CD8^+^ IC   - Low infiltration - Intermediate infiltration - High infiltration | 37 (56.1)  11 (16.7)  18 (27.3) | 39 (54.9)  16 (22.5)  16 (22.5) | 0.63 |
| Stromal infiltration of CD8^+^ IC   - Low infiltration - Intermediate infiltration - High infiltration | 11 (16.7)  28 (42.4)  27 (40.9) | 18 (25.7)  17 (24.3)  35 (50.0) | 0.071 |
| Stromal infiltration of CD4^+^ IC   - Low infiltration - Intermediate infiltration - High infiltration | 16 (24.2)  19 (28.8)  31 (47.0) | 15 (21.7)  17 (24.6)  37 (53.6) | 0.74 |
| Stromal infiltration of CD3^+^ IC   - Low infiltration - Intermediate infiltration - High infiltration | 23 (34.8)  26 (39.4)  17 (25.8) | 26 (37.7)  23 (33.3)  20 (29.0) | 0.76 |

Abbreviations BCT biopsy taken before chemotherapy; ACT biopsy taken after last line of chemotherapy; N number; IC immune cells.
