# Supplementary material for: Association of tumour and stroma PD-1, PD-L1, CD3, CD4 and CD8 expression with DCB and OS to nivolumab treatment in NSCLC patients pre-treated with chemotherapy
Source: Br J Cancer. 2020 May 20;123(3):392–402. doi: 10.1038/s41416-020-0888-5 (PMC7403301; doi:10.1038/s41416-020-0888-5)
Supplement: Supplementary file 2 — Supplementary figures [file 41416_2020_888_MOESM2_ESM.pptx]

## Slide 1
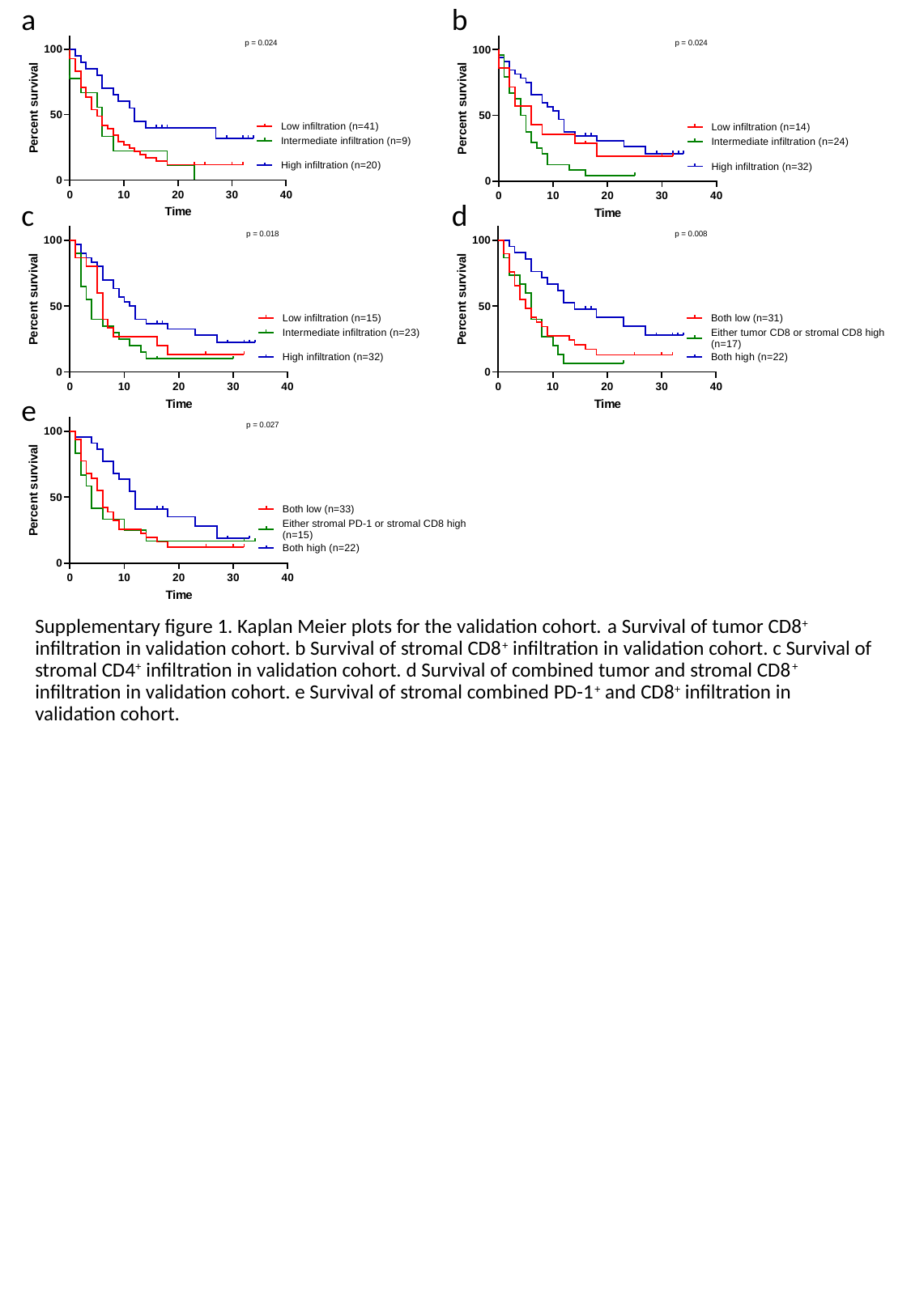

a
b
c
d
e
Supplementary figure 1. Kaplan Meier plots for the validation cohort. a Survival of tumor CD8+ infiltration in validation cohort. b Survival of stromal CD8+ infiltration in validation cohort. c Survival of stromal CD4+ infiltration in validation cohort. d Survival of combined tumor and stromal CD8+ infiltration in validation cohort. e Survival of stromal combined PD-1+ and CD8+ infiltration in validation cohort.

## Slide 2
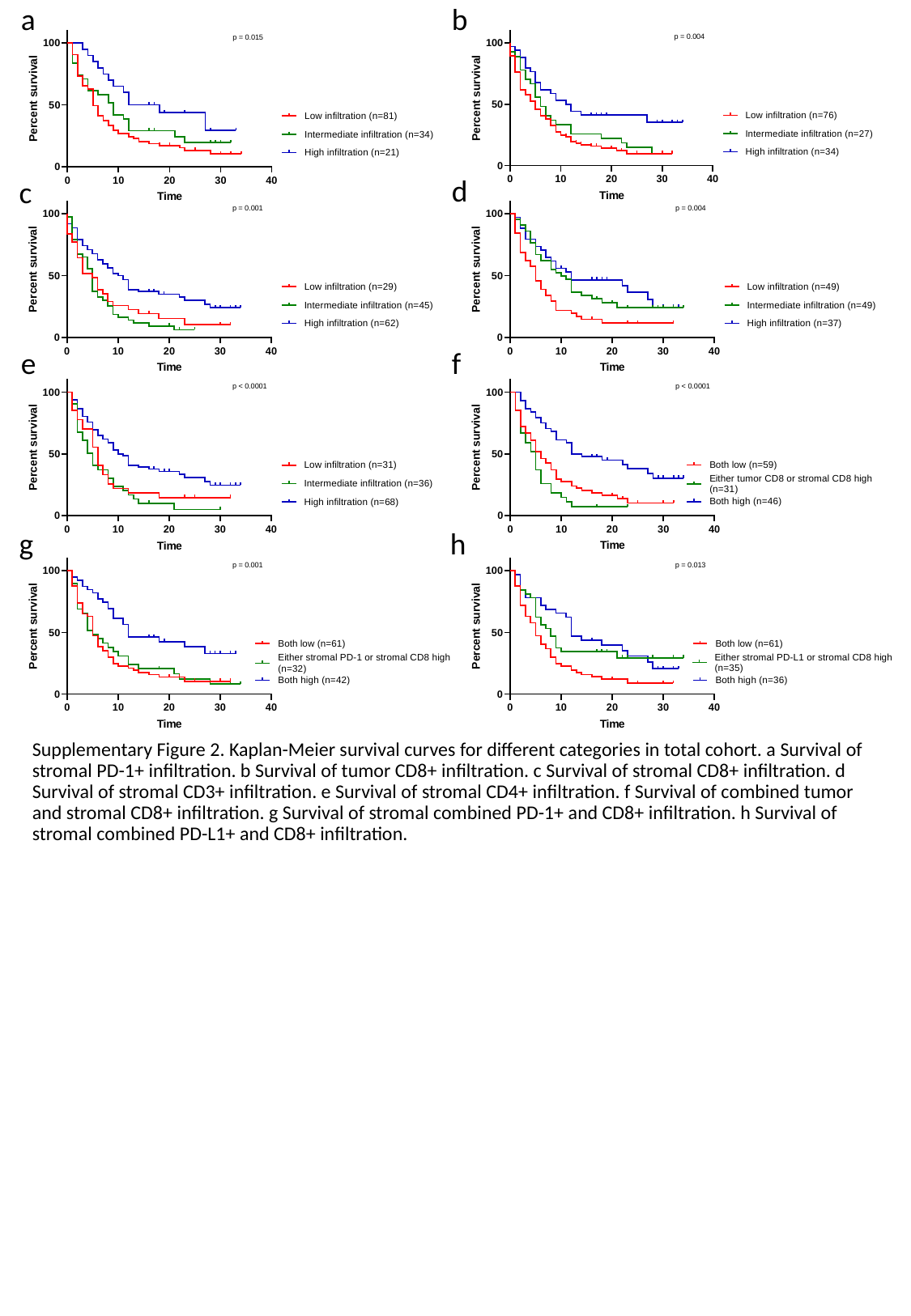

a
b
d
c
f
e
h
g
Supplementary Figure 2. Kaplan-Meier survival curves for different categories in total cohort. a Survival of stromal PD-1+ infiltration. b Survival of tumor CD8+ infiltration. c Survival of stromal CD8+ infiltration. d Survival of stromal CD3+ infiltration. e Survival of stromal CD4+ infiltration. f Survival of combined tumor and stromal CD8+ infiltration. g Survival of stromal combined PD-1+ and CD8+ infiltration. h Survival of stromal combined PD-L1+ and CD8+ infiltration.

## Slide 3
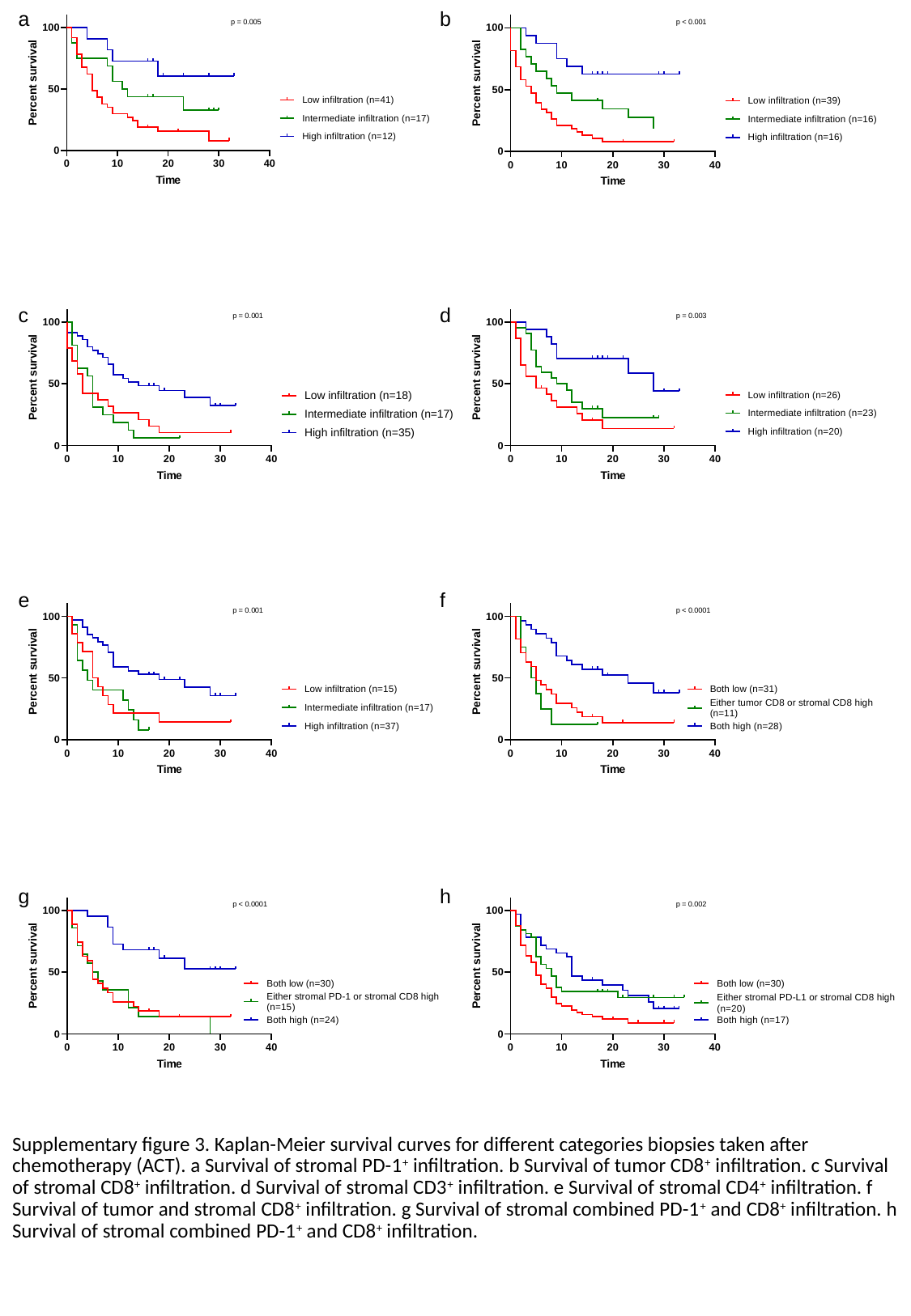

a
b
c
d
e
f
g
h
Supplementary figure 3. Kaplan-Meier survival curves for different categories biopsies taken after chemotherapy (ACT). a Survival of stromal PD-1+ infiltration. b Survival of tumor CD8+ infiltration. c Survival of stromal CD8+ infiltration. d Survival of stromal CD3+ infiltration. e Survival of stromal CD4+ infiltration. f Survival of tumor and stromal CD8+ infiltration. g Survival of stromal combined PD-1+ and CD8+ infiltration. h Survival of stromal combined PD-1+ and CD8+ infiltration.
